# Supplementary material for: Learning curve and surgical outcome of robotic assisted colorectal surgery with ERAS program
Source: Sci Rep. 2022 Nov 29;12:20566. doi: 10.1038/s41598-022-24665-w (PMC9709162; doi:10.1038/s41598-022-24665-w)
Supplement: Supplementary file 2 — Supplementary Information 2. [file 41598_2022_24665_MOESM2_ESM.zip › RAL Raw data-Ñ[▒K/078/HS_ñw╝╨░O▒Kñσ.pdf]

正式摘要(電子簽章：000000215970341)		
W95 / W95 ☐ 英文標題(注意:無英文姓名-請補病人基本檔!)	☐ 【讀取】憑證	

臺中榮民總醫院
出院病歷摘要

轉入號 : 轉出號 :

[redacted]

**(8)**流水編號	
**(9)**入院日期	110/04/22 (2021) 直腸外科 W95 - 12
**(10)**轉科日期	
**(11)**出院日期	110/05/03 (2021) 住院天數計 11 日

診斷

- 入院 :1. functional constipation
- 出院 :1. functional constipation, s/p Robot Assisted Total Colectomy on 04/24

主訴

- Constipation for 30+ year

病史

- .This 51 year old female had history of uterine leiomyoma 5.5 cm
  She suffered from constipation since 17 year-old. She followed up constipation at our CRS OPD since 2013, and recto digital examination showed relaxation of puborectalis was ok, and colon transit study showed value of geometric center are lower than normal
  reference at 48 hours with diagnosis of colon inertia. She visited our CRS OPD on 2021 due to symptom progressed. Colon transit study showed geometric centers of activity at 24-hr and 48-hr are smaller than the reference range, suggestive of prolonged colon transit. Endocrine survey with thyroid and electrolyte showed normal. Under impression of functional constipation, she was admitted to our ward for surgical intervention.

體檢發現

- Anal inspection:
  Recto digital examination:
  Sigmoidoscopy:
  Anoscopy:
  Abdomen:
  - General appearance :
    - easy looking , conscious clear , without cardiopulmonary distress
    - Vital signs自動帶入date 體溫(BT) 脈搏(P) 呼吸(R) 血壓(BP) 身高(BH) 體重(BW) BMI
      20210422 36.2C 75/min 20/min 91/83mmHg 158.7cm 67.2kg 26.7
    - Vital signs: B.P. - mmHg , P.R. 5 /min , R.R. 5 /min
  - HEENT :
    - Eyes: no anemic conjunctiva, no icteric sclera, normal EOM
    - ENT: no otorrhea, no rhinorrhea, no erythematous mucosa, no injected tonsil, no deformity
  - Neck: supple, freely movable, trachea midline,no deformity, no engorged jugular vein , no palpable thyroid or lymph node enlargement
  - Chest:
    - Inspection: Grossly normal, symmetric and fully expansion, no accessory muscle used, no operation scar, no spider angiomata, no gynecomastia
    - Palpation: no palpable mass or nodule, no subcutaneous emphysema, no tenderness
    - Ausculation: clear breathing sound
  - Heart:
    - Percussion: normal heart size
    - Ausculation: regular heart beat, normal S1, S2, no gallop, no murmur

- - Abdomen:
    - Inspection: in
      - flat contour, symetrically, no scar, no stria, no dilated veins, no rashes
    - Ausculation: normoactive bowel sound, no abdominal bruits
    - palpation: soft, no muscle guarding or rigidity, no local tenderness, no rebounding pain, no palpable mass, no palpable pulsation
    - Percussion: no shifting dulllness, no hepatomegaly, no splenomagaly
  -

Peripheral pulsation :	CA	BA	RA	FA	PA	PTA	DPA
Right side :	++	++	++	++	++	++	++/+++
Left side :	++	++	++	++	++	++	++/+++

  - Genito-anus: no tenderness or palpable mass lesion or rectal shelf, normal external genitalia, normal anal tone, brown stool

CRS CONDITION

  -
    - Lung

手術日期及方法

- 手術日期及方法20210423 1. Robotic (XI) assisted subtotal colectomy
  2. Intraoperative colonoscopy : (-).
  3. High ligation (-) : The root was not resected.
  4. Take down from the splenic flexure : (+)
  5. Take down from the hepatic flexure : (+)
  5. OpenGIA (80-3.8) X1 were applied to complete the side-to-end anastomosis
  6. Proximal cut end: > 10 cm, distal cut end: 1.5 cm
  7. No drainage tube was inserted
  8. ADHESION BARRIER FILM was placed
  9. Loop ileostomy (About 15~20cm from IC valve) was done over RLQ of abdomen
  # Notice : While firing the second staple of the robotic endoGIA, malfunction was detected. Special device was applied to release the GIA. A new instrument was used instead, under the safety concern. The third robotic endoGIA was then fired successfully.

住院治療經過

- After admission, pre-op survey was done and there was no contraindication for operation. Robotic assisted total colectomy was performed smoothly on 2021/04/23 and she tolerated the procedure. After op, she recovered fair and poor oral intake was noted for easy abdominal bloating. Medication for symptom was applied and her symtpom was improving gradually. Under stable condition, she was discharge and arranged OPD followed up.

合併症

一般檢查

- 累積報告 :

DATE	TIME	ALB	TP	BIL.T	BIL.D	ALKP	AST	ALT	LDH	CHOL	TG	HDL-C	LDL	UA	NH3	AMY
1100427	0849	3.4		0.6				13								
1100503	0850			0.40			86	125								

DATE	TIME	WBC	RBC	HGB	HCT	MCV	PLT	BLST	NEUT	BAND	SEG	LYM	MON	EOS	BAS
1100427	0858	8520	4.85	12.9			329		82.7			7.7	7.4	1.8	0.4
1100503	0944	6320	3.94	10.4	32.1	81.5	365		67.1			19.1	8.7	4.3	0.8

DATE	TIME	NA	K	CL	CA	BUN	CREAT	Mg	P	CRP	ESR	PCT	LIPASE	LACTATE
1100427	0849	141	3.7		8.2	11	0.62							
1100430	0858	138	3.2	104	7.8		0.53							
1100503	0850	137	4.1			8	0.60							

特殊檢查

病理報告

- 活體組織切片病理報告　手術日期：**110/04/24**　病理號：**S1013687**

  診斷醫師:詹以吉 報告日期:110/04/28
  Pathologic diagnosis:
  Colon, terminal ileum, and appendix, robotic total colectomy --- a.

- Submucosal congestion and lymphoid hyperplasia, of terminal ileum and colon. No tumor seen. b. Fibrous obliteration of appendix.
  Gross description:
  The specimen consists of a segment of colon, 65 cm in length, and 6 cm in proximal circumference, 4 cm in distal cut end circumference, and a segment of terminal ileum, 4 cm in length, and 2.5 cm in cut end circumference. An unremarkable appendix, 5x0.4 cm, is noted. Some erythromatous change over the mucosa is noted and no mass lesion is identified grossly. The appendix, ileocecal valve, and both cut ends are free. Representative part for section: A) proximal cut end B) distal cut end C) ileocecal valve D) appendix E-H) colon mucosa.
  #T-59420_2 #M-36100_2 0 1105 000000
  #T-59200_2 #M-78250_2 0 1105 000000

  細胞病理報告　手術日期：**110/04/22**　病理號：**C1013039**

  診斷醫師:楊啟順 報告日期:110/04/26
  CYTOPATHOLOGICAL DIAGNOSIS:
  Vagina-cervix --- Negative for intraepithelial lesion or malignancy.
  ADEQUACY:
  Satisfactory for interpretation,
  two slides fixed in 95% alcohol.
  MICROSCOPIC DESCRIPTION:
  Mature squamous cells present.
  COMMENT:
  There is possibility of false negative result of cervical/vaginal smear. It is still necessary for regular follow-up if the smear result is negative.
  #T-EA563_2 #M-09414_2 1 3110 000001

其它

出院時情況

- 3.改門診治療

出院指示

藥名	劑量	劑量單位	用法	頻次	數量	數量單位	現況	天數	單位
$Biofermin tab	3	TAB	PO	TID	72	TAB	TH	8	天
Ultracet tab	0.5	TAB	PO	Q6H	16	TAB	TH	8	天
$Miyarisan BM powd.	1	PACK	PO	TID	24	PACK	TH	8	天
Dobecon tab 100mg	1	TAB	PO	TIDAC	24	TAB	TH	8	天
Smecta powder 3g	1	PKG	PO	BIDPRN	16	PKG	TH	8	天

出院計劃

主治醫師_2062F 蔣鋒帆_ / 住院醫師_9760F 趙英如_

正式摘要(電子簽章：**000000215970341)**

W95 / W95

doctorRank:B
